# On Modeling Eavesdropping Attacks in Underwater Acoustic Sensor Networks †

**DOI:** 10.3390/s16050721

**Published:** 2016-05-18

**Authors:** Qiu Wang, Hong-Ning Dai, Xuran Li, Hao Wang, Hong Xiao

**Affiliations:** 1 Faculty of Information Technology, Macau University of Science and Technology, Macau; qiu_wang@foxmail.com (Q.W.); lxrget@163.com (X.L.); 2 Big Data Lab, Faculty of Engineering and Natural Sciences, Norwegian University of Science & Technology in Aalesund, 6009 Aalesund, Norway; hawa@ntnu.no; 3 Faculty of Computer, Guangdong University of Technology, Guangzhou 510006, China; wh_red@gdut.edu.cn

**Keywords:** security, eavesdropping, underwater acoustic sensor networks, isotropic hydrophones, array hydrophones

## Abstract

The security and privacy of underwater acoustic sensor networks has received extensive attention recently due to the proliferation of underwater activities. This paper proposes an analytical model to investigate the eavesdropping attacks in underwater acoustic sensor networks. Our analytical framework considers the impacts of various underwater acoustic channel conditions (such as the acoustic signal frequency, spreading factor and wind speed) and different hydrophones (isotropic hydrophones and array hydrophones) in terms of network nodes and eavesdroppers. We also conduct extensive simulations to evaluate the effectiveness and the accuracy of our proposed model. Empirical results show that our proposed model is quite accurate. In addition, our results also imply that the eavesdropping probability heavily depends on both the underwater acoustic channel conditions and the features of hydrophones.

## 1. Introduction

With the proliferation of various underwater applications such as underwater environment monitoring, offshore structural health monitoring and target tracking, underwater acoustic sensor networks (UASNs) have received much attention. Due to the high attenuation of electromagnetic waves in underwater environments, acoustic communications are typically used in underwater sensor networks. Recently, security has become one of the important concerns in UASNs [1]. We summarize related works on security of UASNs as follows.

### 1.1. Related Works

There are a number of studies on UASNs. In particular, a review of the early history of the acoustic underwater communication was presented in [2]. Research advances and challenges in underwater sensor networks with acoustic communications were summarized in [3]. Generally, the studies on UASNs can be mainly divided into three categories. The first category focused on analyzing the performance of UASNs [4,5,6,7]; the second category focused on improving the network performance in Medium Access Control (MAC) layer [8,9,10]; the third category focused on designing routing schemes [11,12,13]. Underwater cognitive acoustic networks were proposed in [14] to improve the spectrum reuse of acoustic communications. However, there are few studies investigating the network security of UASNs. In particular, a novel secure scheme was proposed to secure the underwater acoustic sensor networks in [15]. The wormhole attack was investigated in [16]. One of vulnerabilities of UASNs was found in [17] that UASNs are suffering from denial-of-service jamming attacks.

On the other hand, as one of the typical security threats in *terrestrial* wireless sensor networks, eavesdropping attacks have received extensive attention recently. It is difficult to detect the eavesdroppers since they just passively wiretap the transmissions without disclosing their existence. Many malicious attacks often follow the eavesdropping activities [18]. Encryption is one of the most commonly used techniques to protect the confidential communications in cellular networks (e.g., Cellular Message Encryption Algorithm [19]), wireless local area networks (e.g., WEP [20], WPA and WPA2 [21]) and wireless personal area networks (WPAN) [22]. However, encryption algorithms may not be feasible to wireless sensor networks since sensor nodes have the energy constraints and computational constraints [23].

In addition to message encryption, there are a number of anti-eavesdropping countermeasures [24,25,26,27,28,29,30,31,32]. In particular, it is shown [24] that to adjust transmitting power can reduce the eavesdropping risk. Besides, [26,27,28,29] found that using directional antennas in wireless sensor networks can enhance the security. Moreover, to deliberately send spurious messages or (dummy data) [25] can also effectively reduce the eavesdropping success rate. Other anti-eavesdropping schemes include the use of defensive jammers [31,32] to protect the normal transmissions.

It is necessary to investigate the eavesdropping behaviors conducted by eavesdroppers since we can offer better protection on the confidential communications if we have a better knowledge on the eavesdroppers [33,34]. For example, we only need to encrypt the communications in the area or the direction that is vulnerable to eavesdropping attacks so that the security cost can be significantly saved.

However, to the best of our knowledge, there is no study on investigating the eavesdropping attacks in UASNs. The previous analytical models on investigating the eavesdropping activities in terrestrial wireless sensor networks [30,35] can not be used to UASNs due to the different features of acoustic signals in underwater environments. For example, the path loss effect of an underwater acoustic channel depends on both the distance and the signal frequency. However, the terrestrial radio channel is independent of the radio signal frequency. Therefore, this is the goal of this paper to establish a novel analytical model and to investigate the eavesdropping activities in UASNs.

### 1.2. Major Contributions

Generally, in conventional UASNs, each node is equipped with an *isotropic hydrophone*, which collects acoustic signals in all directions. We call such underwater acoustic sensor networks with isotropic hydrophones as *IUSNs*. Compared with an isotropic hydrophone, an *array hydrophone* can collect acoustic signals on desired direction. In other undesired directions, there are no signal or weakened signal. Thus, using array hydrophones in UASNs can potentially reduce the interference and consequently improve the network performance. For example, it is shown in [36] that using array hydrophones in UASNs can significantly improve the network throughput. We call such underwater acoustic sensor networks with array hydrophones *AUSNs*. In this paper, we investigate the eavesdropping activities in both IUSNs and AUSNs. We also consider two cases in which an eavesdropper is equipped with an isotropic hydrophone and an array hydrophone, respectively. We name an eavesdropper with an isotropic hydrophone as an isotropic eavesdropper and name an eavesdropper with an array hydrophone as an array eavesdropper.

Our major research contributions in this paper can be summarized as follows.

- We formally propose an analytical model to investigate the probability of eavesdropping attacks in both IUSNs and AUSNs with consideration of underwater acoustic channel conditions, including signal attenuation and ambient noise. In particular, we establish the relationship between the *eavesdropping success condition* and the underwater acoustic signal channel. We further derive the eavesdropping probability with consideration both isotropic eavesdropper and array eavesdropper, respectively.
- We conduct extensive simulations to validate the effectiveness and the accuracy of our proposed model. The simulation results match the analytical results, indicating that our proposed model is accurate.
- We compare the eavesdropping probability of IUSNs and AUSNs. In particular, we find that the eavesdropping probability of AUSNs is lower than that of IUSNs, implying that using array hydrophones in UASNs can reduce the eavesdropping probability. We also find that an array eavesdropper has a higher eavesdropping probability than an isotropic eavesdropper in both IUSNs and AUSNs.
- We find that the eavesdropping probability heavily depends on the acoustic signal frequency, spreading factor, wind speed and the node density. Our results pave the way for designing a better protection mechanism in UASNs.

The rest of this paper is organized as follows. Section 2 first presents the channel model in underwater acoustic communications. We then introduce the transducer used in this paper in Section 3. We next analyze the eavesdropping attacks in UASNs in Section 4. Section 5 gives the empirical results with comparison of IUSNs and AUSNs considering two kinds of eavesdroppers. Finally, we conclude the paper in Section 6.

## 2. Underwater Acoustic Channel Model

In this section, we first instroduce the attenuation of underwater acoustic signal in Section 2.1, and then present the impacts of the ambient noise in Section 2.2.

### 2.1. Attenuation

The attenuation in underwater acoustic communications is characterized by a path loss that depends not only on the distance between the transmitter and the receiver, but also on the signal frequency. In particular, the path loss or the attenuation that occurs in an underwater acoustic channel over a distance *d* for a signal of frequency *f* is given by [37]:

(1) $$A\left(d,f\right)=d^{k}\alpha {\left(f\right)}^{d}$$

where *k* is the spreading factor (ranging from 1 to 2) and α(f) is the absorption coefficient of signal frequency *f*. Note that the different values of *k* describe the different scenarios of geometry of propagation: k=1 for cylindrical spreading; k=2 for spherical spreading.

Usually, Equation (1) can be expressed in dB as follows:

(2) 10logA(d,f)=k×10logd+d×10logα(f)

Generally, if the frequency *f* is above a few hundred Hz, the absorption coefficient 10logα(f) in dB/km for *f* in kHz can be expressed as follows:

(3) $$\begin{array}{c} 10\log\alpha \left(f\right)=0.11\times \frac{f^{2}}{1+f^{2}}+44\times \frac{f^{2}}{4100+f^{2}}\\ +2.75\times 10^{-4}f^{2}+0.003 \end{array}$$

For the lower frequency *f*, we have the absorption coefficient 10logα(f) as follows [38]:

(4) $$\;10\log\alpha \left(f\right)=0.002+0.11\times \frac{f^{2}}{1+f^{2}}+0.011f^{2}$$

### 2.2. Ambient Noise

The ambient noise in underwater acoustic channel is more complicated than that in terrestrial wireless channel. We usually model the ambient noise by four kinds of sources: turbulence, shipping activities, waves, and thermal noise. Specifically, the noise caused by shipping activities can be modeled by shipping activity factor *s*, whose value ranges from 0 to 1 representing the intensity of shipping activities (0 representing low and 1 representing high). The noise of waves, aroused by wind, can be described by wind speed *w* (m/s). In UASNs, the ambient noise depends on signal frequency *f* and the formula between power spectral density of the four noise sources and frequency in kHz in dB re *μ* Pa per Hz are given by [38].

(5) $$\begin{array}{cc} & 10\log N_{t}\left(f\right)=17-30\log\left(f\right)\\ & 10\log N_{s}\left(f\right)=40+20\left(s-0.5\right)+26\log\left(f\right)-60\log\left(f+0.03\right)\\ & 10\log N_{w}\left(f\right)=50+7.5w^{1/2}+20\log\left(f\right)-40\log\left(f+0.4\right)\\ & 10\log N_{th}\left(f\right)=-15+20\log\left(f\right) \end{array}$$

where $N_{t}\left(f\right)$, $N_{s}\left(f\right)$, $N_{w}\left(f\right)$ and $N_{th}\left(f\right)$ denote the noise sources caused by turbulence, shipping activity, wind and thermal, respectively.

Then, the total noise in dB is as follows:

(6) $$10\log N\left(f\right)=10\log(N_{t}\left(f\right)+N_{s}\left(f\right)+N_{w}\left(f\right)+N_{th}\left(f\right))$$

**Figure 1:**
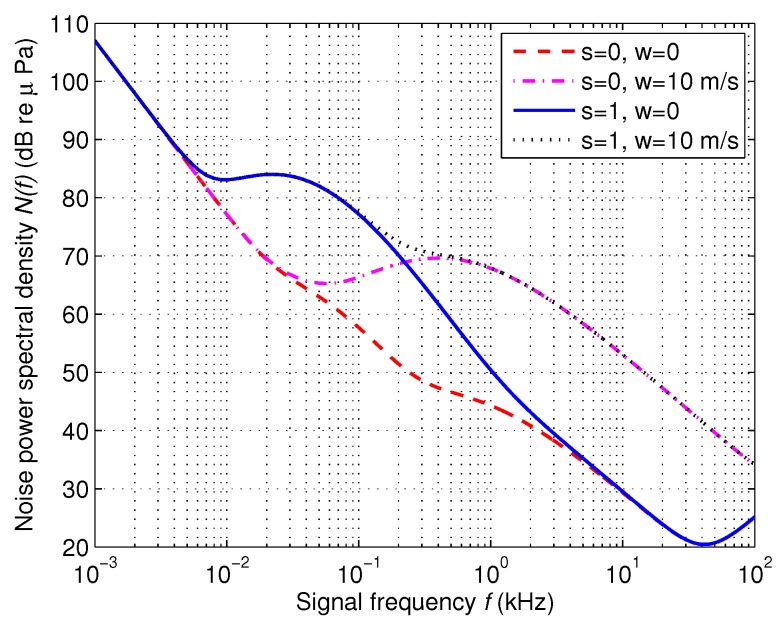
Power spectral density of the ambient noise N(f) (dB re *μ* Pa).

Figure 1 shows the power spectral density of the ambient noise N(f)(dB) in various values of shipping activity *s* and wind speed *w*. It can be seen from Figure 1 that shipping activity factor *s* mainly affects the noise when 0.001<f<1 kHz, while wind speed *w* mainly affects the noise when f>1 kHz, which is the frequency band usually used in underwater communications. Therefore, we will consider the impacts of wind speed *w* on the eavesdropping activities in this paper.

## 3. Transducers

In UASNs, transducers are not only used to implement the conversion of acoustic energy into electrical energy or vice versa, but also used to transmit/receive acoustic signals. Transducers used as receivers are called hydrophones, and those used for transmitting are called transmitters or projectors [39]. In this paper, we focus on hydrophones. Conventional UASNs are commonly equipped with a single isotropic hydrophone which collects acoustic signals uniformly in all directional. Different from a single isotropic hydrophone, modern transducers are comprised of an array of hydrophones which receive signals more effectively in some directions (*i.e.*, improving signal noise ratio in some directions). Moreover, with the evolution of VLSI, modern hydrophones are able to form beams in desired directions. Thus, this section will introduce these two types of hydrophones.

**Figure 2:**
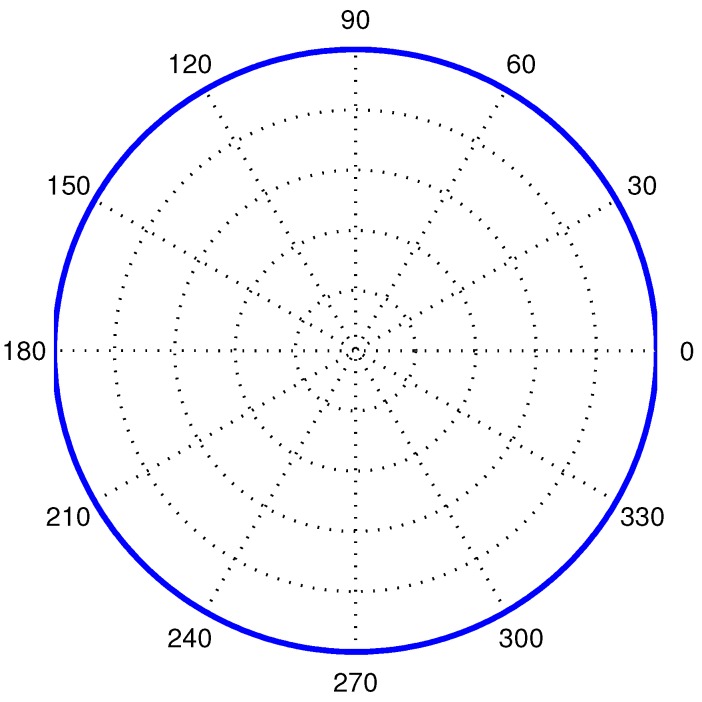
The beam pattern of an isotropic hydrophone.

An isotropic hydrophone collects acoustic signal uniformly in all directions. In particular, Figure 2 shows the beam pattern of an isotropic hydrophone.

An array hydrophone consists of an array of isotropic hydrophones. To model the characteristics of an array hydrophone, we introduce *Array Gain*, which is the improved gain on signal-to-noise ratio (SNR) compared with an isotropic hydrophone. The array gain can be expressed as follows [39]:

(7) $$AG=\frac{\sum_{i=1}^{M}\sum_{j=1}^{M}a_{i}a_{j}\beta_{s}^{ij}}{\sum_{i=1}^{M}\sum_{j=1}^{M}a_{i}a_{j}\beta_{n}^{ij}}$$

where *M* is the number of isotropic hydrophone elements of an array hydrophone, $a_{i}$ and $a_{j}$ are the amplitude shading coefficients of the *i*-th hydrophone element and the *j*-th hydrophone element, respectively. If array hydrophones are unshaded, $a_{i}=a_{j}=1$. $\beta_{s}^{ij}$ is the correlation of the received signals between the *i*-th hydrophone element and the *j*-th hydrophone element and $\beta_{n}^{ij}$ is the correlation of the noise between the *i*-th hydrophone element and the *j*-th hydrophone element.

**Figure 3:**
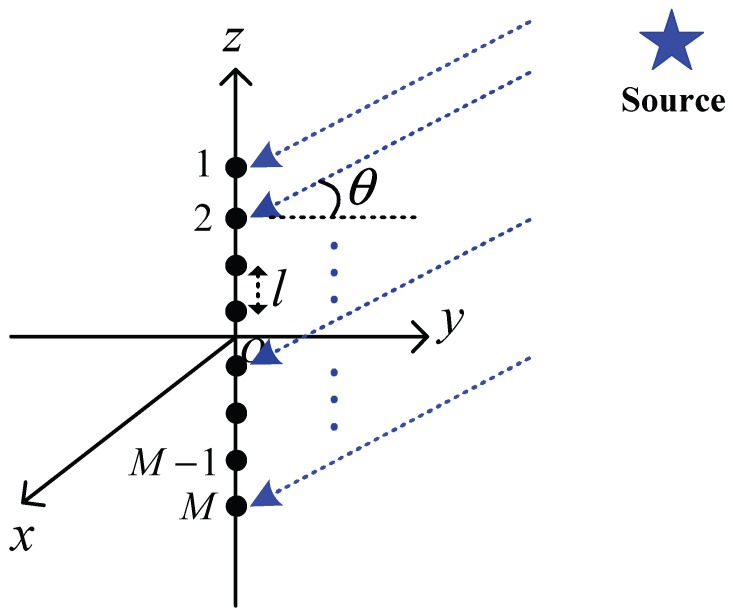
A line array hydrophone with *M* isotropic hydrophone elements.

One of most commonly used array hydrophones is an unshaded uniform linear array transducer, which consists of *M* isotropic hydrophones evenly spaced in a line, as shown in Figure 3. In this structure, *l* is the distance between two neighboring elements, and *λ* is the wavelength of acoustic wave radiated from signal source (denoted by the blue star in Figure 3). In underwater communication, a signal wave is usually considered as a plane wave, meaning that signals can be received by each hydrophone element in the same direction *θ*. Although plane waves cannot be generated in practice, both spherical and cylindrical waves approximate plane waves when they are sufficiently far from signal source [39]. Therefore, we consider acoustic signal as a plane wave in this paper.

Given this unshaded uniform linear array hydrophone, $\beta_{s}^{ij}$ is described as follows:

(8) $$\beta_{s}^{ij}=\cos\left(\frac{2\pi }{\lambda }\left|i-j\right|l\sin\theta \right)$$

where $\left|\cdot \right|$ denotes the absolute value.

In this paper, we consider that the array hydrophone uses a location identification scheme to acquire the direction of signal source and then adjust their angles to transmitters. Thus, we have θ=0. Then we apply this value in Equation (8) and obtain the correlations of received signal $\beta_{s}^{ij}=1$.

In general, we consider omni-directional noise, meaning that noise surround each hydrophone with constant power in all directions. Then, the correlations of received noise $\beta_{n}^{ij}$ is expressed by

(9) $$\beta_{n}^{ij}=\left\{\begin{array}{cc} 1 & i=j\\ \frac{\sin(\frac{2\pi }{\lambda }\left|i-j\right|l)}{\frac{2\pi }{\lambda }\left|i-j\right|l} & i\neq j \end{array}\right.$$

**Figure 4:**
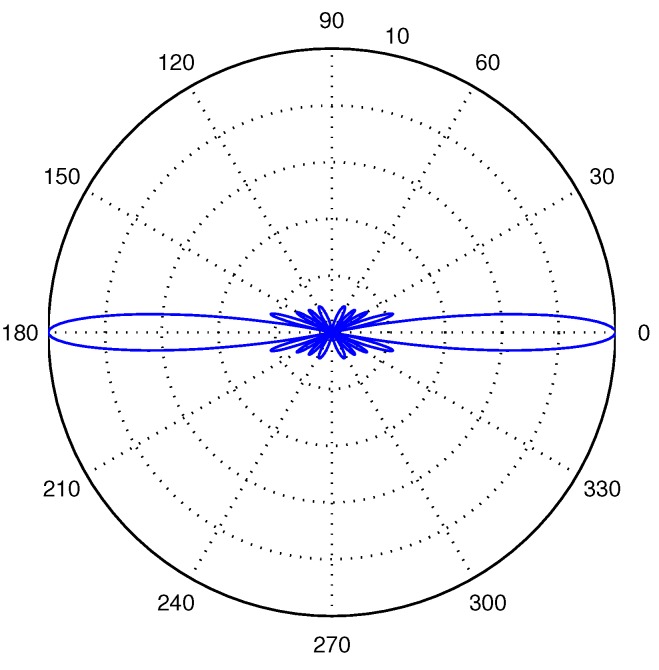
The beam pattern of an unshaded uniform liner array hydrophone with 10 isotropic hydrophone elements.

A common choice of the distance between the two neighboring isotropic hydrophone elements is $l=\frac{\lambda }{2}$. Applying the value in Equations (8) and (9) and combining with Equation (7), we then get AG=M. This means that an unshaded uniform linear array hydrophone can improve *M* times of signal-to-noise ratio compared to a single isotropic hydrophone, which is an important factor used in our analysis. The beam pattern of an unshaded uniform linear array hydrophone with 10 elements is shown in Figure 4.

## 4. Analysis of Eavesdropping Attacks in UASNs

In our eavesdropping model, we consider two scenarios of UASNs according to the two different types of hydrophones: Scenario (i), in which every node is equipped with a single isotropic hydrophone; Scenario (ii), in which every node is equipped with an unshaded uniform linear array hydrophone. Scenario (i) and Scenario (ii) correspond to IUSNs and AUSNs, respectively. In addition, we consider that all the nodes are randomly distributed in a 2-D plane according to a homogeneous Poisson point process with density *ρ* in both IUSNs and AUSNs. Then, we consider two types of eavesdroppers: isotropic eavesdroppers and array eavesdroppers in both IUSNs and AUSNs. Recall that an isotropic eavesdropper is equipped with a single isotropic hydrophone and an array eavesdropper is equipped with an unshaded uniform linear array hydrophone. Thus, we investigate the impacts of different eavesdroppers on the eavesdropping probability of IUSNs and AUSNs, respectively.

Before we analyze eavesdropping activities, we have to analyze link criteria in both IUSNs and AUSNs in Section 4.1 and then derive eavesdropping success condition in Section 4.2. We next derive the eavesdropping probability in Section 4.3.

### 4.1. Link Criteria

We first define the link condition in UASNs. We denote the transmission power by $P_{t}$. The signal-to-noise ratio at receiver denoted by SNR can be calculated according to the aforementioned underwater acoustic channel model in Section 2 as follows:

(10) $$SNR=AG\frac{P_{t}}{\int_{B}A\left(d,f\right)df\int_{B}N\left(f\right)df}$$

where *B* is the bandwidth used in acoustic communications. In order to investigate the relationship between frequency *f* and SNR better, we normalize the bandwidth. Then the Equation (10) can be simplify to

(11) $$SNR=AG\frac{P_{t}}{A(d,f)N(f)}$$

Equation (11) is a general equation in both IUSNs and AUSNs. But note that in IUSNs, AG=1, while in AUSNs, AG=M. Equation (11) can be expressed in terms of dB by $SNR_{dB}$ as

(12) $$SNR_{dB}=10\log AG+10\log P_{t}-10\log A\left(d,f\right)-10\log N\left(f\right)$$

Generally, a link can be successfully established if and only if

(13) $$SNR_{dB}\geq \Delta_{0}\left(dB\right)$$

where $\Delta_{0}$ is the minimum signal-to-noise ratio at receiver. In particular, we let the signal-to-noise ratio equal to $\Delta_{0}$, and then have the minimum transmission power $P_{t}$ in dB

(14) $$10\log P_{t}=\Delta_{0}+10\log A\left(d,f\right)+10\log N\left(f\right)-10\log AG$$

We denote the minimum transmission power in IUSNs by $P_{t}^{i}$ and that in AUSNs by $P_{t}^{a}$. Then, we can express the relationship between $P_{t}^{i}$ and $P_{t}^{a}$ from Equation (14) with different value of AG as

(15) $$10\log P_{t}^{a}=10\log P_{t}^{i}-10\log M$$

In this paper, we assume that both AUSNs and IUSNs apply power control scheme to save power. In other words, the nodes in AUSNs and IUSNs use the minimum transmission power $P_{t}^{i}$ and $P_{t}^{a}$, respectively. We can see from Equation (15) that the transmitters in AUSNs can use less transmission power to establish link under the same network topology and environment conditions compared with the transmitters in IUSNs.

### 4.2. Eavesdropping Success Condition

We now define the eavesdropping success condition that determines whether an eavesdropper can successfully wiretap the communication between a transmitter and a receiver. It is obvious that the eavesdropper can tap information implying that the eavesdropper can establish a link with a node. Thus, the eavesdropper can successfully tap information if and only if it fulfill Equation (13). Combining Equations (2), (12) and (13), we can have

(16) $$k\times 10\log d+d\times 10\log\alpha \left(f\right)+10\log N\left(f\right)-10\log AG\leq 10\log P_{t}-\Delta_{0}$$

where AG=1 when networks with an isotropic eavesdropper and AG=M when networks with an array eavesdropper. We observe that left-hand-side (LHS) of Equation (16) is an increasing function of the distance *d*. If we let LHS of Equation (16) be equal to right-hand-side (RHS), we then can obtain the *maximum eavesdropping distance* $d_{\max}$, within which an eavesdropper can wiretap a transmission successfully.

Generally, it is *non-trivial* to obtain the exact expression of $d_{\max}$ since Equation (16) is a transcendental function of *d*. However, we observe from Equation (16) that it is not difficult to obtain the numerical results of $d_{\max}$ when the frequency *f*, environment parameters (spreading factor *k*, and wind speed *w*) and the transmission power $P_{t}$ are given.

**Figure 5:**
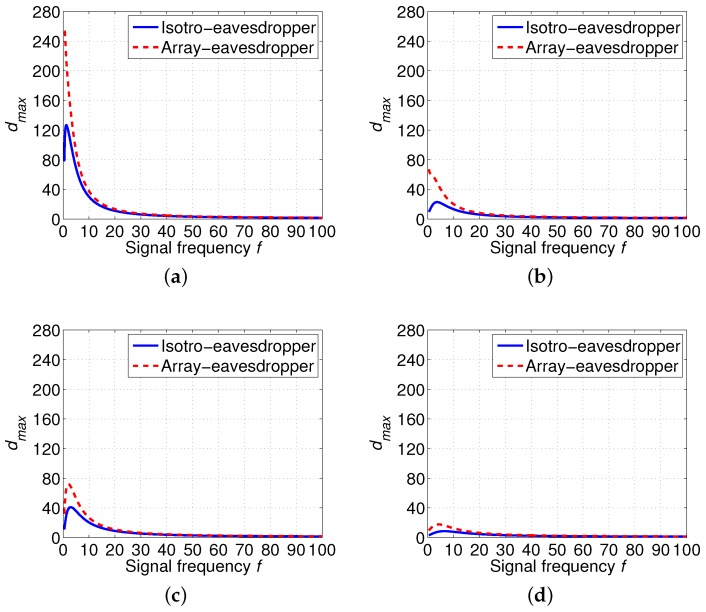
Maximum eavesdropping distance $d_{\max}$ (km) according to different signal frequency *f* (kHz) based on different spreading factor *k* and wind speed *w* when $P_{t}=100\;\mathrm{dB}$, $\Delta_{0}=20\;\mathrm{dB}$. (**a**) k=1,w=0m/s; (**b**) k=1,w=10m/s; (**c**) k=2,w=0m/s; (**d**) k=2,w=10m/s.

Thus, we calculate $d_{\max}$ with different frequency *f*, *k* and *w* with the threshold of signal-to-noise $\Delta_{0}=20\;\mathrm{dB}$ [38] and $P_{t}=100$ dB considering an isotropic eavesdropper and an array eavesdropper (M=10), respectively. The numerical results are presented in Figure 5. Specifically, we can see from Figure 5 that an isotropic eavesdropper (red dash lines) have higher $d_{\max}$ than an array eavesdropper (blue solid lines). Besides, $d_{\max}$ varies with different signal frequency *f* when environment parameters (spreading factor *k*, and wind speed *w*) are fixed. Furthermore, as shown in Figure 5, when the spreading factor *k* is fixed, increasing wind speed *w* leads to decreasing $d_{\max}$. This is the result of the higher noise when wind speed *w* is higher. It can also be seen from Figure 5 that with the fixed wind speed *w*, $d_{\max}$ decreases with the increased spreading factor *k*. The reason is that increasing spreading factor *k* leads to higher absorption of the acoustic signal.

### 4.3. Eavesdropping Probability

**Figure 6:**
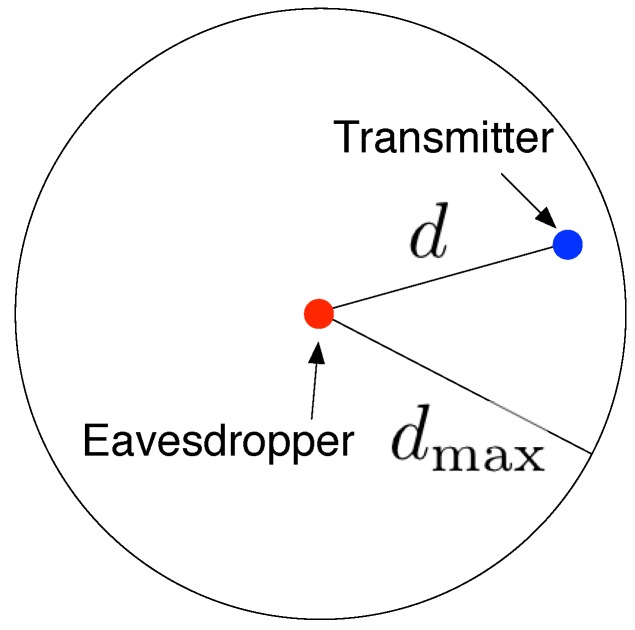
Eavesdropping region is a circle with radius $d_{\max}$.

We model the successful chance of eavesdropping attacks by the *eavesdropping probability*, denoted by P(E). To derive P(E), we need to calculate the probability of no node being eavesdropped first. We then consider an *effective eavesdropping area D*, which is the expected value of the critical region where the communication can be wiretapped by eavesdroppers only when a node falls in this region. Since the eavesdropper is equipped with a single isotropic hydrophone, it wiretaps acoustic signals uniformly in all directions, as shown in Figure 6. In particular, we have

(17) $$D=\pi d_{\max}^{2}$$

where the value of $d_{\max}$ can be calculated by Equation (16).

We next denote the number of nodes in the eavesdropping area by a random variable *Y*. Since nodes are randomly distributed in a 2-D area according to a homogeneous Poisson point process, we then have the probability of no node falling in the eavesdropping area, which is given by

(18) $$P\left(Y=0\right)=e^{-\rho D}$$

Substituting *D* in Equation (18) by RHS of Equation (17), we finally obtain the *eavesdropping probability* P(E) by the following equation:

(19) $$P\left(E\right)=1-P\left(Y=0\right)=1-e^{-\rho \pi {\left(d_{\max}\right)}^{2}}$$

From the aforementioned analysis, the higher spreading factor *k* and the higher wind speed *w*, the shorter the maximum eavesdropping distance $d_{\max}$ is. Therefore, we can see from Equation (19) that with the fixed node density *ρ*, the higher spreading factor *k* and the higher wind speed *w*, the lower probability of eavesdropping attacks P(E) is. Our empirical results will further confirm this observation.

## 5. Empirical Results

In this section, we conduct extensive simulations to evaluate the accuracy and the effectiveness of our proposed analytical model. As indicated in the previous work [40], the border effect often affects the accuracy of the simulation results. In this paper, we consider a simplified approach to eliminate the border effect. Specifically, we place the eavesdropper at the center of simulation area, which is large enough so that the border effect can be ignored.

The statistical eavesdropping probability denoted by $P_{s}\left(E\right)$ is calculate by the following equation

(20) $$P_{s}\left(E\right)=\frac{\mathrm{No}.\mathrm{of}\mathrm{topologies}\mathrm{that}\mathrm{have}\mathrm{been}\mathrm{eavesdropped}}{\mathrm{No}.\mathrm{of}\mathrm{topologies}}$$

The physical meaning of Equation (20) is the percentage of the number of topologies being eavesdropped to the total number of topologies. As shown in the previous work [40], when the total number of topologies →∞, we can accurately approximate the theoretical result. In practice, we generate 10,000 random topologies for each run of simulations in this paper (to balance the accuracy against the computational complexity).

Then, we conduct extensive simulations by dividing them into two groups according to impacts of different eavesdropper: isotropic eavesdropper and array eavesdropper (M=10). In each group, we investigate the eavesdropping probability in IUSNs and AUSNs, respectively. Note that we assume both IUSNs and AUSNs have the same network topologies and environment conditions with a fixed threshold of signal-to-noise threshold $\Delta_{0}=20\;$dB.

### 5.1. Eavesdropping Probability with an Isotropic Eavesdropper

We first conduct the first group of simulations with consideration of an isotropic eavesdropper in IUSNs and AUSNs, respectively.

#### 5.1.1. Eavesdropping Probability in IUSNs

**Figure 7:**
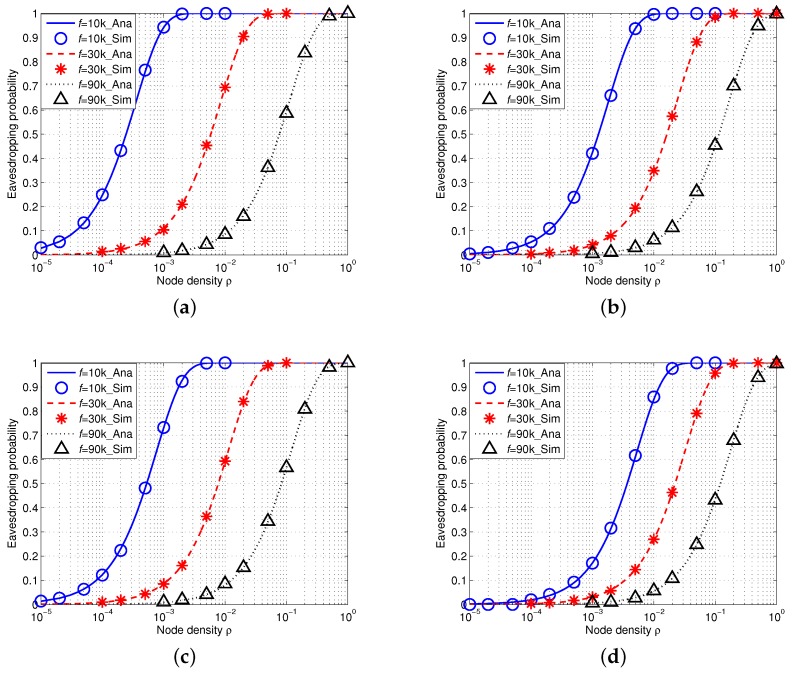
Eavesdropping probability in IUSNs with an *isotropic* eavesdropper versus node density *ρ* (/km${}^{2}$) when f=10 kHz, f=30 kHz and f=90 kHz under different spreading factor *k* and wind speed *w*. (**a**) k=1,w=0m/s; (**b**) k=1,w=10m/s; (**c**) k=2,w=0m/s; (**d**) k=2,w=10m/s.

Figure 7 shows the simulation results of eavesdropping probability in IUSNs with an isotropic eavesdropper. We assign the acoustic signal frequency *f* with 10 kHz, 30 kHz and 90 kHz under different values of spreading *k* and wind speed *w* and we fix the reference transmission power $P_{t}^{i}=100\;\mathrm{dB}$. In particular, the analytical results are shown as curves and the simulation results are shown as markers. We can see from Figure 7 that there is an excellent agreement of the simulation results with the analytical results. This indicates that our proposed analytical model is accurate. Besides, as shown in each curve of Figure 7, the eavesdropping probability increases with the node density *ρ*. This phenomenon can be explained by Equation (19), *i.e.*, the more nodes, the higher the chance of being eavesdropped.

Furthermore, we compare the results with different values of frequency f=10kHz, f=30kHz and f=90kHz, denoted by the blue, red and black curves, respectively. More specifically, we find that the eavesdropping probability decreases with the increased values of frequency *f*. This is because the acoustic signal attenuation goes much faster with the increased signal frequency in underwater environment when *f* is high (*i.e.*, f>8kHz). Moreover, Figure 7 also shows that the eavesdropping probability decreases when wind speed *w* increases or spreading factor *k* increases. This trend confirms our aforementioned observation in Section 4.

#### 5.1.2. Eavesdropping Probability in AUSNs

**Figure 8:**
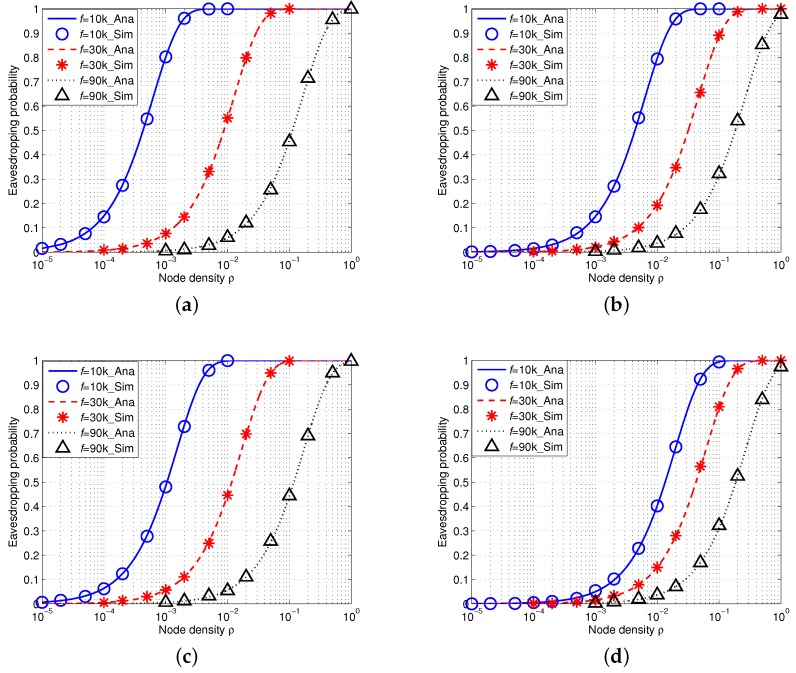
Eavesdropping probability in AUSNs with an *isotropic* eavesdropper versus node density *ρ* (/km${}^{2}$) when f=10 kHz, f=30 kHz and f=90 kHz under different spreading factor *k* and wind speed *w*. (**a**) k=1,w=0m/s; (**b**) k=1,w=10m/s; (**c**) k=2,w=0m/s; (**d**) k=2,w=10m/s.

We then conduct simulations on the eavesdropping probability in AUSNs with an array eavesdropper (M=10). Figure 8 shows the results. Note that the transmission power in AUSNs can be calculated by Equation (15) and the reference transmission power is $P_{t}^{i}=100\;\mathrm{dB}$. Similarly, we can also draw a conclusion that our analytical framework can accurately model the eavesdropping probability in AUSNs as the simulation results match the analytical results. Besides, we can see that the eavesdropping probability increases with the increased node density *ρ* and decreases with the increased signal frequency *f*, and eavesdropping probability decreases when wind speed *w* or spreading factor *k* increases. Moreover, if we compare the eavesdropping probability in IUSNs with that in AUSNs together (by aligning Figure 7 and Figure 8 together), we can find AUSNs have the lower values in terms of eavesdropping probability than IUSNs, implying that the nodes have less chance of being eavesdropped upon in AUSNs than that in IUSNs.

### 5.2. Eavesdropping Probability with an Array Eavesdropper

We then conduct another group of simulations with consideration of an array eavesdropper in IUSNs and AUSNs, respectively.

#### 5.2.1. Eavesdropping Probability in IUSNs

**Figure 9:**
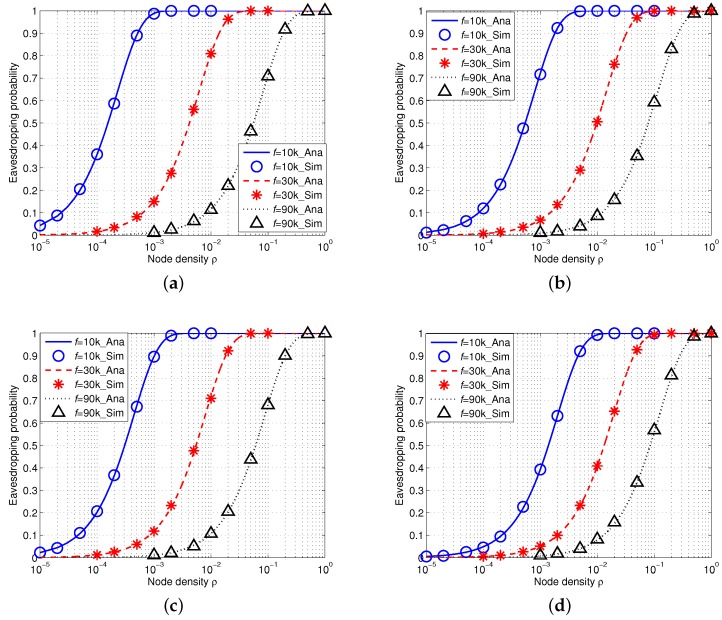
Eavesdropping probability in IUSNs with an *array* eavesdropper versus node density *ρ* (/km${}^{2}$) when f=10 kHz, f=30 kHz and f=90 kHz under different spreading factor *k* and wind speed *w*. (**a**) k=1,w=0m/s; (**b**) k=1,w=10m/s; (**c**) k=2,w=0m/s; (**d**) k=2,w=10m/s.

Figure 9 shows the simulation results of eavesdropping probability in IUSNs with an array eavesdropper. There is an excellent agreement of the simulation results with the analytical results, further confirming the accuracy of our model. Similarly, as shown in Figure 9, the eavesdropping probability increases with the node density *ρ* and decreases with the increased values of frequency *f*. Figure 9 also shows that the eavesdropping probability decreases when wind speed *w* increases or spreading factor *k* increases.

#### 5.2.2. Eavesdropping Probability in AUSNs

**Figure 10:**
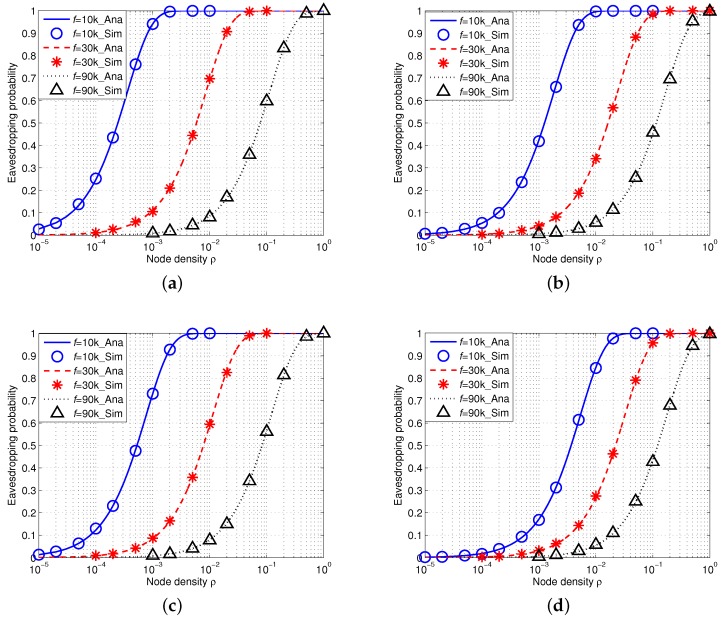
Eavesdropping probability in AUSNs with an *array* eavesdropper with different nodes density *ρ* (/km${}^{2}$) when f=10 kHz, f=30 kHz and f=90 kHz under different spreading factor *k* and wind speed *w*. (**a**) k=1,w=0m/s; (**b**) k=1,w=10m/s; (**c**) k=2,w=0m/s; (**d**) k=2,w=10m/s.

Figure 10 shows the results of eavesdropping probability in AUSNs with an array eavesdropper (M=10). Similarly, their transmission power can be calculated by Equation (15) and the reference transmission power of nodes is $P_{t}^{i}=100$ dB. We can also see that the simulation results accurately match the analytical results. Besides, the eavesdropping probability increases with the increased node density *ρ* and decreases with the increased signal frequency *f*. Moreover, the eavesdropping probability decreases with increased wind speed *w* or spreading factor *k*. If we align Figure 9 and Figure 10 together, we can find AUSNs have the lower eavesdropping probability than IUSNs, which implies that nodes have less chance of being eavesdropped upon in AUSNs than in IUSNs.

### 5.3. Comparison between an Isotropic Eavesdropper and an Array Eavesdropper

If we compare the eavesdropping probability of an isotropic eavesdropper with that of an array eavesdropper in AUSNs by aligning Figure 8 and Figure 10 together, we can see that an array eavesdropper always has the higher eavesdropping probability than an isotropic eavesdropper in AUSNs. We have a similar result in the case by comparing Figure 7 and Figure 9 in IUSNs.

## 6. Conclusions

In this paper, we propose an analytical model to investigate the eavesdropping probability in underwater acoustic sensor networks, which have different channel characteristics from those of terrestrial wireless sensor networks. In particular, we first establish the relationship between the eavesdropping probability and the underwater acoustic channel in both IUSNs and AUSNs considering an isotropic eavesdropper and an array eavesdropper, respectively. We then conduct extensive simulations to validate our model. The simulation results match our analytical results implying that our model is accurate and effective at analyzing the eavesdropping probability in underwater acoustic sensor networks. Besides, we also find that the eavesdropping probability heavily depends on signal frequency. Moreover, the results also show that the eavesdropping probability increases with the increased node density, decreases with the increased wind speed and decreases with the increased spreading factor. Comparing the eavesdropping probability in IUSNs with that in AUSNs, we find that equipping nodes with array line hydrophones can significantly decrease the eavesdropping probability of underwater acoustic sensor networks. One of the future research directions is to evaluate the effectiveness of our analytical model in realistic test-beds though it is challenging to implement underwater acoustic sensor networks (even with isotropic hydrophones) [41].
